# Epileptogenic Zone Localization in Refractory Epilepsy by FDG-PET: The Comparison of SPM and SPM-CAT With Different Parameter Settings

**DOI:** 10.3389/fneur.2021.724680

**Published:** 2021-10-07

**Authors:** Eric Jacob Bacon, Chaoyang Jin, Dianning He, Shuaishuai Hu, Lanbo Wang, Han Li, Shouliang Qi

**Affiliations:** ^1^College of Medicine and Biological Information Engineering, Northeastern University, Shenyang, China; ^2^Key Laboratory of Intelligent Computing in Medical Image, Ministry of Education, Northeastern University, Shenyang, China; ^3^Department of Neurosurgery, Shengjing Hospital of China Medical University, Shenyang, China; ^4^Department of Radiology, Shengjing Hospital of China Medical University, Shenyang, China

**Keywords:** refractory epilepsy (RE), cortical dysplasia, epileptogenic zone (EZ), FDG-PET, SPM

## Abstract

Refractory epilepsy is a complex case of epileptic disease. The quantitative analysis of fluorodeoxyglucose positron emission tomography (FDG-PET) images complements visual assessment and helps localize the epileptogenic zone (EZ) for better curative treatment. Statistical parametric mapping (SPM) and its computational anatomy toolbox (SPM-CAT) are two commonly applied tools in neuroimaging analysis. This study compares SPM and SPM-CAT with different parameters to find the optimal approach for localizing EZ in refractory epilepsy. The current study enrolled 45 subjects, including 25 refractory epilepsy patients and 20 healthy controls. All of the 25 patients underwent surgical operations. Pathological results and the postoperative outcome evaluation by the Engel scale were likewise presented. SPM and SPM-CAT were used to assess FDG-PET images with three different uncorrected *p*-values and the corresponding cluster sizes (k), as in voxels in the cluster, namely *p* < 0.0002, *k* > 25; *p* < 0.001, *k* > 100; *p* < 0.005, and *k* > 200. When combining three settings, SPM and SPM-CAT yielded overall positive finding scores of 96.0% (24/25) and 100.0% (25/25) respectively. However, for the individual setting, SPM-CAT achieved the diverse positive finding scores of 96.0% (24/25), 96.0% (24/25), and 88.0% (22/24), which are higher than those of SPM [88.0% (22/25), 76.0% (19/25), and 72.0% (18/25)]. SPM and SPM-CAT localized EZ correctly with 28.0% (7/25) and 64.0% (16/25), respectively. SPM-CAT with parameter settings *p* < 0.0002 and *k* > 25 yielded a correct localization at 56.0% (14/25), which is slightly higher than that for the other two settings (48.0 and 20.0%). Moderate concordance was found between the confirmed and pre-surgical EZs, identified by SPM-CAT (kappa value = 0.5). Hence, SPM-CAT is more efficient than SPM in localizing EZ for refractory epilepsy by quantitative analysis of FDG-PET images. SPM-CAT with the setting of *p* < 0.0002 and *k* > 25 might perform as an objective complementary tool to the visual assessment for EZ localization.

## Introduction

Epilepsy is among the most common neurological disorders affecting people of all ages. It is characterized by unpredictable seizures and can give rise to other health problems. Recent statistics indicate that epilepsy affects more than 50 million people worldwide (1). Refractory epilepsy is a drug-resistant epilepsy; patients are considered to suffer from refractory epilepsy if disabling seizures continue despite treatment trials with two anti-seizure drugs, either alone or in combination (2). Diagnosing refractory epilepsy remains a tedious task. While several researchers investigated refractory epilepsy to diagnose and reveal possible causes (3, 4), the main cause remains unknown, and doctors are yet to determine why some patients are receptive to medicine and others not.

Advances in neuroimaging continue to improve the surgical treatment of refractory epilepsy (5). Powerful neuroimaging techniques have been developed to make the diagnosis straightforward. Among these techniques, the fluorodeoxyglucose positron emission tomography (FDG-PET) has shown particular efficiency during the pre-surgical evaluation. It exhibits high sensitivity in detecting the epileptogenic zone (EZ) of cortical dysplasia (CD), which is known to occur in refractory epilepsy patients. Hence, FDG-PET contributes to localizing seizure onset zone (SOZ) in epilepsy surgery (6, 7). It has furthermore demonstrated high sensitivity to detect hyper-metabolic areas in patients with refractory epilepsy (8).

Although FDG-PET is to date a promising imaging modality technique in detecting the EZ, its visual assessment may lack accuracy, as its sensitivity is estimated to span 35–86% (9–11). However, visual interpretation can be improved by applying further analysis (12).

Advanced tools or software can be used for the improvement of visual interpretation of FDG-PET. A typical example is voxel-based morphometry (VBM), whose pipeline follows a standard procedure that includes brain tissue segmentation, spatial normalization, registration, and smoothing. During VBM procedures, the changes in gray matter (GM) and white matter (WM) in individual patients are evaluated. One of the most common tools used when performing VBM is statistical parametric mapping (SPM). This tool has revealed its effectiveness in the EZ localization (13). Computational Anatomy Toolbox 12 (CAT12) is a toolbox of SPM12 and it can be used to perform VBM through SPM^1^. SPM-CAT performs better than SPM by efficiently identifying brain morphological abnormalities in patients with temporal lobe epilepsy (TLE) (14). SMP-CAT is assumed to be more accurate in localizing the EZ than SPM; however, no such report is available.

Pre-surgical evaluation using FDG-PET images is necessary for refractory epilepsy, improving accurate EZ localization and providing better surgery outcomes. However, the tool's performance and how to set the appropriate parameters for SPM and SPM-CAT are unknown. This study aims to compare SPM and SPM-CAT with different parameter settings and find the appropriate localizing EZ in refractory epilepsy by FDG-PET images. The performance of each approach and setting has been first compared to each other, and subsequently the identified pre-surgical EZ was compared to the confirmed EZ according to the postsurgical follow-up. To the best of our knowledge, no studies have been performed for such an evaluation using both VBM approaches with different settings.

## Materials and Methods

### Materials

#### Participants

**Figure 1:**
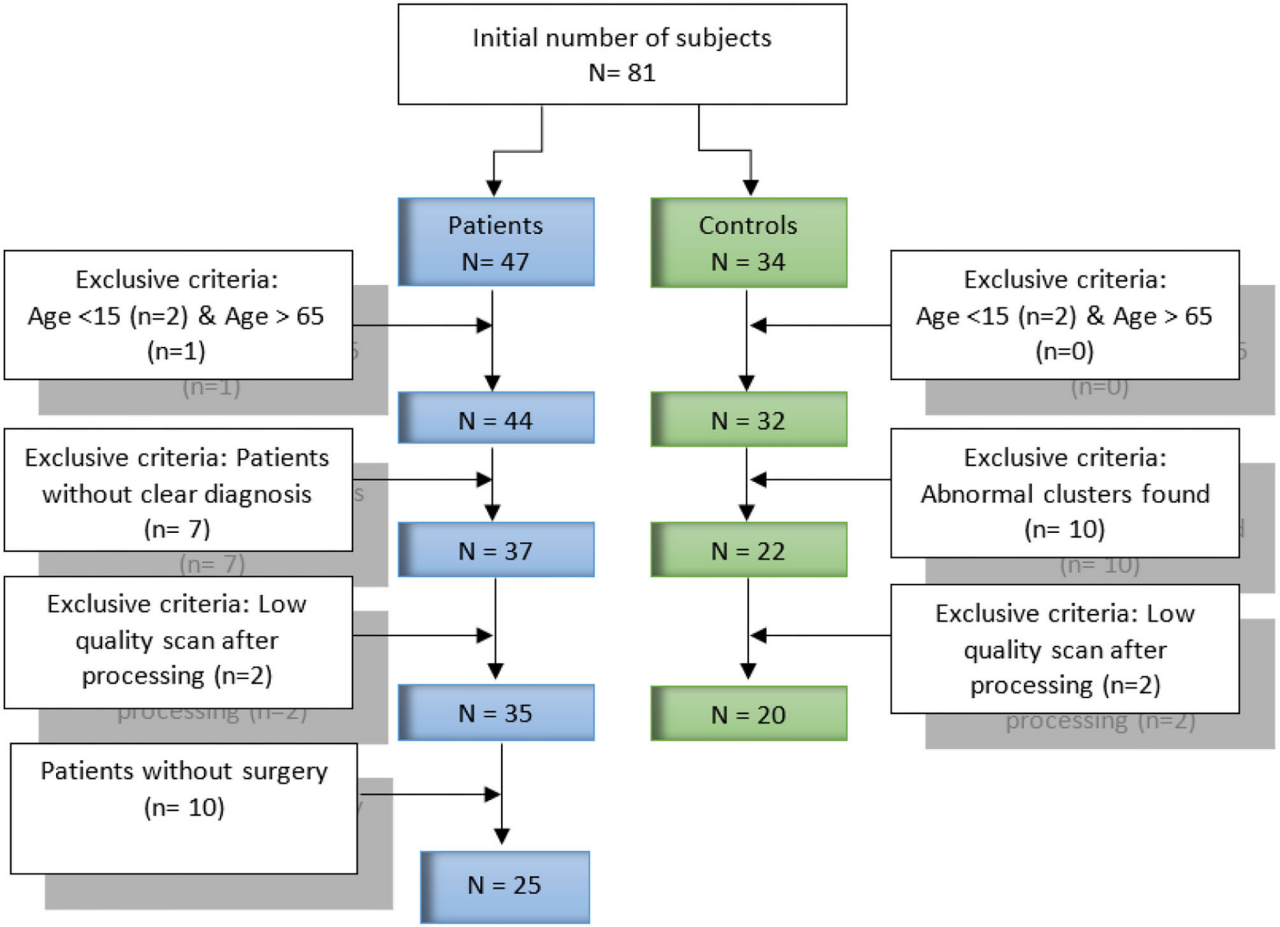
Criteria and candidate selection procedure.

Our dataset contains data collected form 81 FDG-PET subjects (47 patients with refractory epilepsy and 34 healthy controls). All subjects and datasets were subjected to some selection criteria, such as age and the obtained image quality, respectively. Figure 1 provides further detail about the selection criteria. In total, 45 subjects were selected for our current study, including 25 patients and 20 healthy controls. The mean age of the patients was 31.1 years [standard deviation (SD), 10.8 years], of which 72.0% (18/25) were male and 28.0% (7/25) female. For healthy controls, the mean age was 25.8 years, SD, 7.7 years, of which 42.9% (8/20) were male and 57.1% (12/20) female. Patients underwent pre-surgical evaluation from January 2018 to July 2019 at Shengjing Hospital of China Medical University (Shenyang, China). The evaluation involved a detailed clinical history and neurological examination, complete neuropsychological evaluation, psychiatric assessment, inter-ictal and ictal onset patterns in long-term scalp video-electroencephalogram (video-EEG), magnetic resonance imaging (MRI), and PET results. Images for both groups of patients and healthy controls were acquired following the clinical routine of epilepsy. The ethics committee of China Medical University's Shengjing Hospital (Shenyang, China) granted their approval to the report. The study protocol was explained to all participants, after which they signed an informed consent form.

In some cases, the epileptic zone can be localized by pre-operative stereoelectroencephalography (SEEG). If this epileptic zone does not include the eloquent area (e.g., motor or language), then this zone will be surgically removed. If the epileptic zone identified by SEEG includes the eloquent area, intraoperative electrocorticography (ECoG) was used to avoid the eloquent area and specify the resection zone. In case SEEG is not required, the epileptic and resection zones were determined by intraoperative ECoG. The electrode with eight contactors is commonly employed in SEEG, while 32 (four rows and eight columns) or 16 contactors (two rows and eight columns) are usually included in ECoG according to the size of the epileptic zone.

For the surgical operation of temporal lobe epilepsy, there is a standard procedure to follow by the surgeons. According to international practice, some standard anatomical marks in both neocortex and medial structure can be referred for the surgical resection. For the surgery of extra-temporal lobe (medial or deep epileptic foci) epilepsy, cerebral gyrus, sulcus and superficial blood vessels should be visualized through multi-modality images, and the epileptic zone should be clearly marked in the pre-operative plan. Meanwhile, intraoperative navigation and localization are required.

Table 1 provides the demographic information of patients and healthy controls. For the 25 patients who underwent surgery, the outcomes were evaluated in terms of the Engel value, and EZ was confirmed by the postsurgical follow-up. The Engel value was defined after 6 months following the surgery. Table 2 gives the detailed semiology of 25 epilepsy patients in this study. This semiology has referred to the 2017 International League Against Epilepsy (ILAE) classification of epilepsies (15).

**Table 1:** Clinical characteristics of epilepsy patients and healthy controls.

**Characteristics**		**Epilepsy patients**	**Healthy controls**
Number of patients		25	20
Gender	Male	18	8
	Female	7	12
Age (years)		15–63	15–48
(mean ± S.D.)		(31.12 ± 10.8)	(25.8 ± 7.7)

**Table 2:** Semiology of epilepsy patients in this study.

**No**.	**Semiology**
1	Hand automatism with impaired awareness, sometimes secondary head/eye versive
2	Autonomic auras, dialeptic seizure
3	Versive with impaired awareness, tachycardia
4	Head/eye versive with impaired awareness, tonic
5	Hand automatism with impaired awareness, versive
6	Oroalimentary and hand automatism with impaired awareness, tonic
7	Hypermotor with impaired awareness
8	Hand automatism with impaired awareness
9	Eyelid fluttering with impaired awareness, nonversive head turning, oroalimentary automatism
10	Hand versive with impaired awareness, tonic
11	Oroalimentary and hand automatism with impaired awareness, versive
12	Tonic with impaired awareness, hand automatism
13	Asymmetric tonic with impaired awareness
14	Psychic aura, oroalimentary automatism without impaired awareness
15	Hand automatism with impaired awareness
16	Oroalimentary and hand automatism with impaired awareness
17	Clonic with impaired awareness, secondary bilateral tonic-clonic
18	Dialeptic seizure
19	Tachycardia, myodystonia with impaired awareness, tonic
20	automatism with impaired awareness
21	Oroalimentary and hand automatism with impaired awareness
22	Tonic
23	Oroalimentary and hand automatism with impaired awareness
24	Gelastic, hypermotor
25	Oroalimentary and hand automatism with impaired awareness

For healthy controls, 32 subjects were retained after the age criteria. We selected each of these 32 subjects and compared it with the others by SPM and SPM-CAT analysis to find abnormal clusters. In case of an abnormal cluster, this subject was excluded. Finally, 24 and 22 patients were retained for SPM and SPM-CAT analysis, respectively. The overlapping 22 subjects were determined as healthy controls for further exclusive criteria. Two subjects with low-quality data were excluded, and 20 subjects were finally retained. This procedure is the same as the one employed by Mayoral et al. (16).

#### PET Data Acquisition

All PET measurements were acquired and processed with a specific epilepsy protocol as used in clinical routine, irrespective of being conducted on the patients or control group. Images of patients were acquired using a PET/MRI scanner (SIGNA PET/MR; GE Healthcare, Waukesha, WI, USA). The subjects were asked to rest quietly in a dimly lit room for about 45–60 min after the intravenous administration of 18F-FDG with 3.7 MBq/kg. The default 3D ordered subsets expectation maximization (OSEM) algorithm (32 subsets and three iterations) was used to reconstruct PET images. The restored data has a 192 × 192 × 16 matrix and a 1.56 × 1.56 × 2.40 mm^3^ voxel scale. The acquisition time of each scan was 15 min.

Images of healthy controls were acquired with the General Electric Discovery 690 PET (GE Medical Systems) in Shengjing Hospital of China Medical University because the PET/MRI scanner was newly installed and no image data of healthy controls is available. After the intravenous administration of ~5 MBq/kg of 18F-FDG, the patients were asked to rest quietly in a dimly lit room for about 40 min. The projection data of 25 tomographic attenuation-corrected brain parts of 3.27-mm thickness were obtained through a standard routine of 11 min. The scan mode was the helical mode with a rescale slope of 1.0 and a reconstruction diameter of 700 mm. PET data were reconstructed using the OSEM algorithm (16 subsets and six iterations). The restored data have a 512 × 512 × 16 matrix and a 3.65 × 3.65 × 3.27 mm^3^ voxel scale.

### Methods

#### Procedure of SPM and SPM-CAT

**Figure 2:**
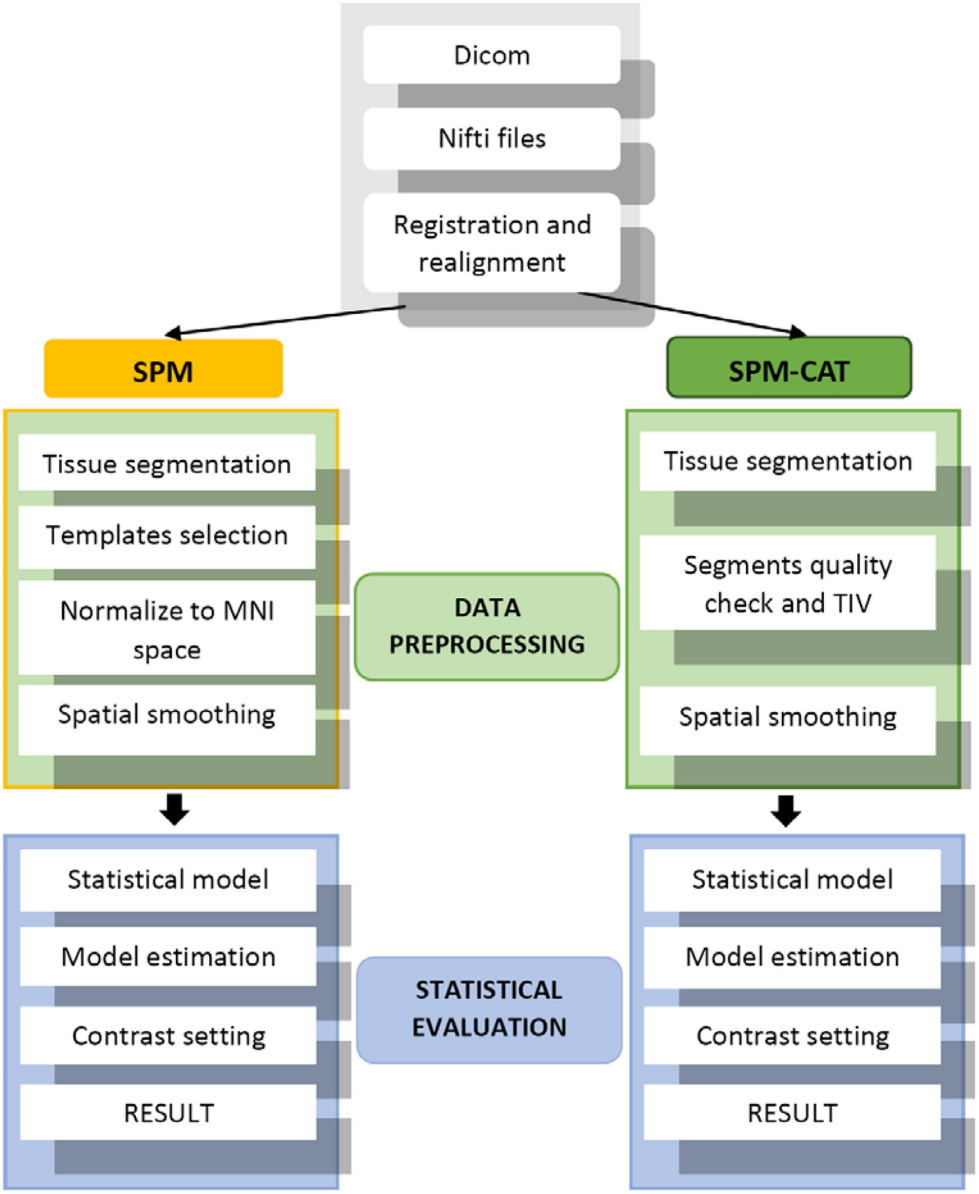
SPM and SPM-CAT procedures.

The procedure used in this study involves applying the VBM pipeline mentioned in (13). In total, data from 25 patients with refractory epilepsy were used in our evaluation. Figure 2 outlines three main steps: (1) data preparation, (2) data processing, and (3) statistical evaluation. For the data preparation, SPM and SPM-CAT have the same procedure. The images in Dicom were first converted into the format of Nifti, and the alignment check-up and registration to canonical templates were followed.

For the data processing of SPM, the brain tissue was first segmented into gray matter (GM), white matter (WM), and cerebrospinal fluid (CSF). Subsequently, the proportional scaling intensity normalization was performed. After selecting the canonical template, the diffeomorphic anatomic registration through an exponentiated lie (DARTEL) algebra algorithm was used to normalize the segmented scans into a standard Montreal Neurological Institute (MNI) space (17). This spatial normalization was only conducted for the segmented GM to find the EZ. Furthermore, the spatially normalized GM was smoothed by a Gaussian kernel of full width at half maximum (FWHM) 8 × 8 × 8 mm^3^. Statistical evaluation was performed as the final step of processing using the SPM model. A two-sample *t*-test comparison with age as a covariate was performed between each patient and the healthy control database using an implicit mask based on a gray-level threshold of 0.3. A digital human brain atlas tool called xjview was used to determine the location of hypo-metabolic areas (18, 19). These hypo-metabolic areas or clusters with the most significant volume are assumed to be the EZ.

SPM-CAT went through the regular VBM pipeline using both SPM and CAT12 GUI to analyze the images. In the data processing of SPM-CAT, the first part is the tissue segmentation performed with CAT GUI. During the tissue segmentation, CAT12 uses the standard tissue probability maps (TPMs) as provided in SPM. The latter dynamically uses the appropriate template for spatial registration, either DARTEL (20) or Geodesic Shooting (21) with a predefined template. As with SPM, only the gray matter was analyzed. The second part of the data processing involved the segment quality check and total intracranial volume estimation. The third part was the spatial smoothing implemented in the same manner as in SPM. Finally, the statistical evaluation was performed using CAT12 GUI and its statistical model. However, the same two-sample *t*-test and settings as in SPM were adopted.

#### Performance Comparison and Statistical Analysis

For each patient, SPM and SPM-CAT were performed with three different parameter settings: *p* < 0.0002, *k* > 25; *p* < 0.001, *k* > 100; and *p* < 0.005, *k* > 200. The uncorrected *p*-value is the statistical threshold specifying the level of variation of FDG activity considered to be significant, while performing the segmentation of statistical parametric maps. k is the predetermined size of the cluster (i.e., the number of voxels in the cluster).

In this study, cluster is defined as a group of voxels. In the case of abnormal clusters, this patient was defined as the “positive study”. The positive finding score was calculated as the percentage or rate of the number of positive studies over the total number of patients (i.e., 25). In SPM, if any of the three-parameter settings reports the positive finding for one patient, we assume this patient as the “overall positive study”. Therefore, the overall positive finding score of SPM can be calculated. This is similar to SPM-CAT.

By using SPM and SPM-CAT with different parameter settings, one or more hypo-metabolic clusters will be identified for each patient. If more than one clusters are identified, as commonly done in previous studies (10, 22), the cluster with the largest volume is defined as the pre-surgical EZ identified by SPM and SPM-CAT.

EZ was identified by SPM and SPM-CAT with the confirmed EZ according to the postsurgical follow-up. The identified EZ location is given as the left or/and right hemisphere and the temporal, frontal, parietal, and occipital lobe. If the identified and the confirmed EZs match, a correct localization is considered to be achieved for this patient. A correct localization percentage can be determined for the 25 patients with surgery.

In SPM, for each patient, if any of the three-parameter settings identifies the EZ matching with the confirmed EZ, we assume that this patient is the “overall correct localization study”. In this manner, we obtain the overall correct localization percentage for SPM. The overall correct localization percentage of SPM-CAT is determined in the same manner.

The positive finding score is compared within three-parameter settings and the overall situation by McNemar's test for SPM and SPM-CAT. The overall positive finding scores of SPM and SPM-CAT were also compared. If *p* < 0.05, a significant difference is available. The same comparison is made for the correct localization percentage.

To compare the identified EZ among different settings, different locations (or lobes) are assigned values from zero to two (0–negative, 1–left hemisphere, 2–right hemisphere). For each setting, one vector of 25 elements will be obtained, and the value of each element will be 0, 1, or 2. For the overall situation, the value of the element will be 1 if the patient is determined as “overall correct localization study”; otherwise, it will be 0. McNemar's test is applied to determine the significance of the differences between different vectors (parameter settings).

A statistical analysis was conducted between the identified EZ with SPM and SPM-CAT with different parameter settings and the confirmed EZ by Cohen's kappa's test. Its 95% confidence interval (CI 95%) was likewise given. The kappa value (*k*) can be interpreted as follows: *k* ≤ 0 indicating no agreement and 0.01–0.20 as none to slight, 0.21–0.40 as fair, 0.41–0.60 as moderate, 0.61–0.80 as substantial, and 0.81–1.00 as almost perfect agreement. All of the statistical analyses were performed using SPSS software, ver. 16.0 (IBM-SPSS, Armonk, NY, USA).

## Results

### Epileptogenic Zone Identified by SPM and SPM-CAT

**Figure 3:**
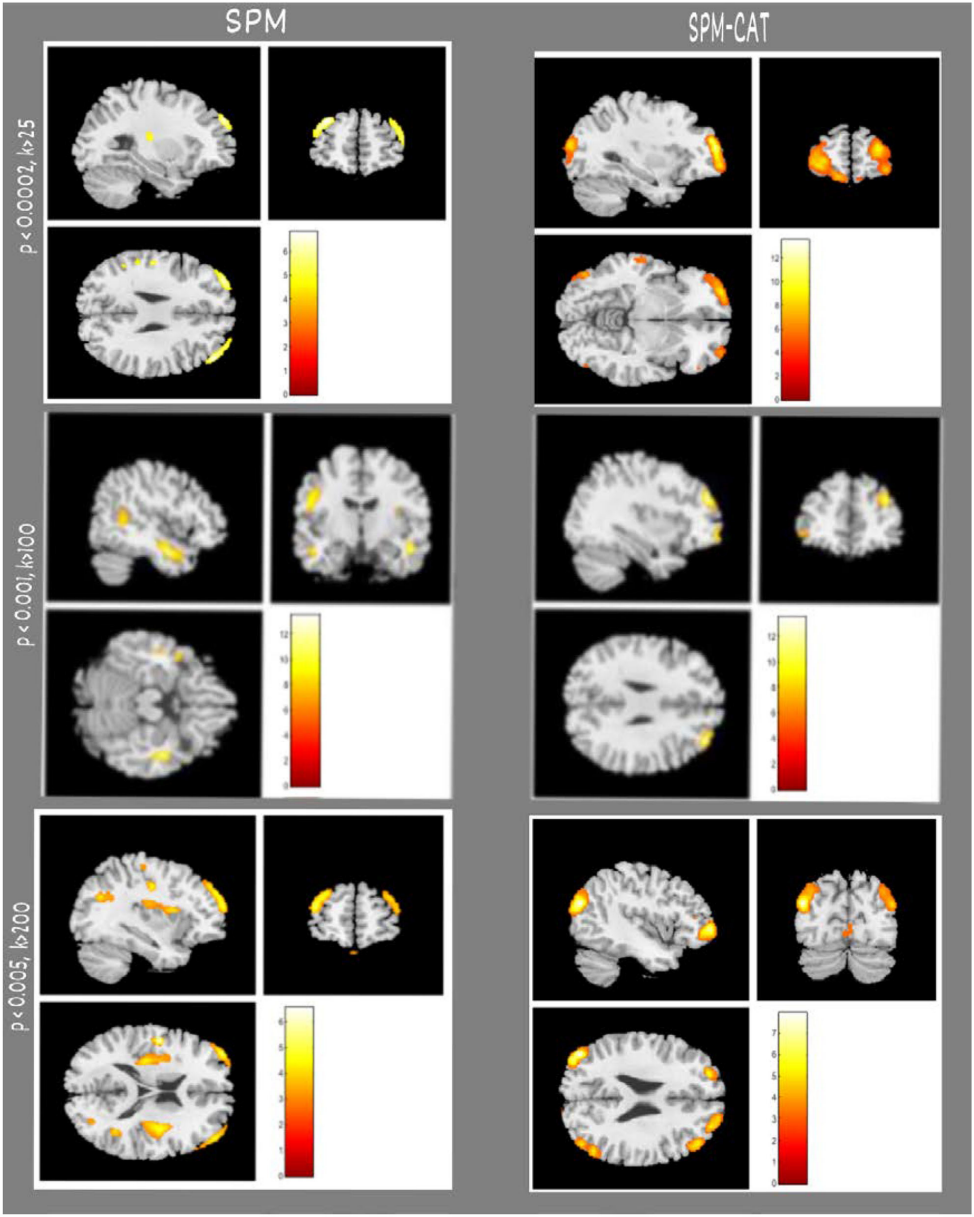
Examples of epileptogenic zones identified by SPM and SPM-CAT in three patients. Each row indicates the results of a patient (e.g., No. 01, 03, 12). From the first to the third row, the parameter settings of SPM and SPM-CAT are *p* < 0.0002, *k* > 25; *p* < 0.001, *k* > 100; *p* < 0.005, and *k* > 200. The left two columns are for SPM with sagittal and coronal views, and the right two columns are for SPM-CAT with sagittal and coronal views.

In the examples shown in Figure 3, SPM and SPM-CAT localized different hypo-metabolic areas through the analysis in three patients (1, 3, and 12) with different parameter settings. Table 3 lists significant metabolic changes in different areas of the brain of three patients (L = left, F = frontal, T = temporal, and li = limbic lobes) for the same three patients as presented in Figure 3. For each patient, the clusters with the largest voxel size have been selected associated with their spatial information (coordinates, peak intensities, voxels size, and brain region) and presented as significant findings. The aim of Figure 3 and Table 3 is to present typical results as examples.

**Table 3:** Comparison of epileptogenic zones identified by SPM and SPM-CAT (cluster with largest volume) in three patients as examples.

**No**.	**SPM**				**SPM-CAT**				**Parameter setting**
	**Brain areas**	**Voxels per cluster**	**Coordinates**	**Peak intensity**	**Brain areas**	**Voxels per cluster**	**Coordinates**	**Peak intensity**	
01	L/F	215	−28.9, 54.0, 28.9	5.2	L/F	402	−33.0, 60.0, −6.0	8.5	*p* < 0.0002, *k* > 25
03	R/T	759	−18.5, −8.8, 47.0	7.3	R/F	204	34.9, 45.0, 28.9	4.3	*p* < 0.001, *k* > 100
12	L/F	1,587	−39.0, 57.0, 18.0	5.6	L/T	2,171	−43.5, −78.0, 22.5	7.7	*p* < 0.005, *k* > 200

### Positive Finding Scores of SPM and SPM-CAT

**Figure 4:**
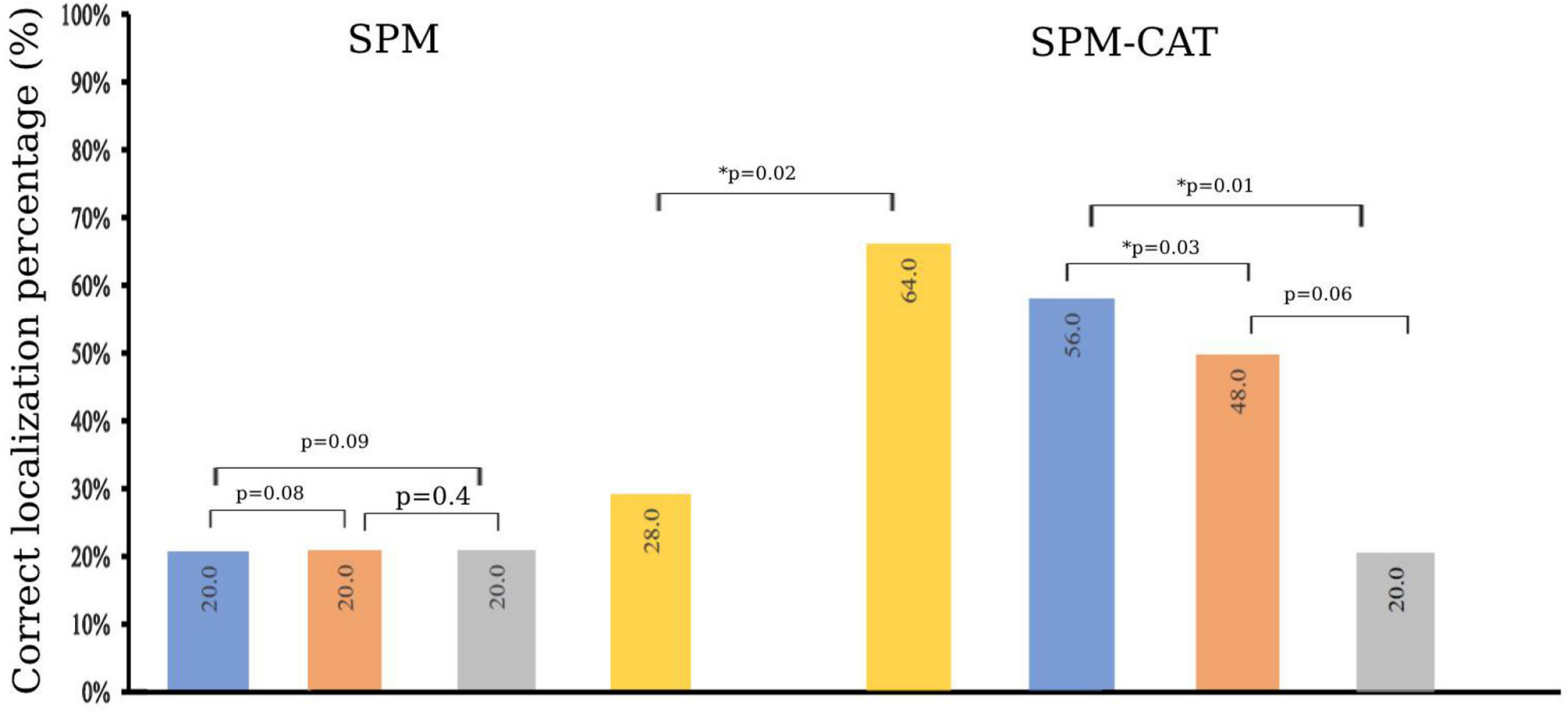
Positive finding score of SPM and SPM-CAT with different parameter settings (*p*-value is from McNemar's test).

The finding score for positives is different for SPM and SPM-CAT with different parameter settings (Figure 4). For the three settings (*p* < 0.0002, *k* > 25; *p* < 0.001, *k* > 100; *p* < 0.005, *k* > 200), the positive finding score was 88.0% (22/25), 76.0% (20/25), and 72.0% (18/25), respectively. There were no significant differences among the three settings (*p* > 0.05, McNemar's test). The overall positive finding score of SPM was 96.0% (24/25) and significantly higher than that of the setting of *p* < 0.0002 and k > 25 (*p* < 0.05, McNemar's test).

The positive finding score of SPM-CAT with the three respective settings was 96.0% (24/25), 96.0% (24/25), and 88.0% (22/25). SPM-CAT has a significantly higher positive finding score for each setting than that of SPM (*p* < 0.05, McNemar's test).

SPM-CAT has a better overall positive finding score than SPM [100.0% (25/25) and 96.0% (24/25) respectively], i.e., the abnormal findings (hypo-metabolic areas) were observed in FDG-PET images of 25 epilepsy patients.

### Correct Localization Percentage of EZ

Table 4 presents the clusters with a significant difference in FDG-PET images identified by SPM and SPM-CAT with different parameter settings, as well as the confirmed EZ according to the postsurgical follow-up, and the outcomes of patients with surgery. For SPM, three different parameter settings generated the same EZ (or negative results) for 17 patients. For the remaining eight patients, one or two settings yielded the negative finding. For SPM-CAT; the same EZ was found only for four patients when using three different settings. The variations among different settings are larger in SPM-CAT than in SPM. Among 25 patients with surgery, 20 have the confirmed EZ at the temporal lobe, three patients at the frontal lobe, one patient at the parietal lobe, and one at both temporal and occipital lobes. The outcomes of patients with surgery were good (Engel I to Engel III). Specifically, Engel I accounted for 68% (17/25), Engel II for 8% (2/25), and Engel III for 24% (6/25).

**Table 4:** Comparison between clusters with a significant difference in FDG-PET images and confirmed EZ according to postsurgical follow-up.

**No**.	**SPM**			**SPM-CAT**			**EZ**	**Engel**
	***p* < 0.0002, *k* > 25**	***p* < 0.001, *k* > 100**	***p* < 0.005, *k* > 200**	***p* < 0.0002, *k* > 25**	***p* < 0.001, *k* > 100**	***p* < 0.005, *k* > 200**		
01	LF	L/F	L/F	L/F	L/T	LF	L/F	1
02	L/F	L/F	L/F	R/T	R/T	L/F	R/mT	2
03	R/T	Neg	Neg	R/F	R/F	R/T	R/mT	3
04	L/F	L/F	L/F	L/T	L/T	L/F	L/mT	1
05	L/li	L/li	L/s-l	R/O	R/O	L/li	R/mT	1
06	L/T	L/T	Neg	R/O	R/O	L/T	R/m&laT	1
07	R/O	Neg	Neg	R/T	R/T	R/O	R/mT	3
08	L/T	Neg	Neg	R/F	R/F	L/T	R/mT	1
09	L/F	L/T	L/F	L/T	L/T	L/F	L/mT	1
10	L/F	L/F	L/F	L/F	L/F	L/F	L/F	1
11	R/O	R/O	R/O	R/F	R/F	R/O	R/mT	1
12	L/F	L/F	Neg	L/T	L/T	L/F	L/mT	1
13	L/F	L/F	L/F	L/O	L/O	L/F	L/mT	2
14	Neg	Neg	Neg	L/T	L/T	Neg	L/mT	1
15	L/li	L/li	L/li	Neg	Neg	L/li	R/P	3
16	L/F	L/F	L/F	L/T	L/T	L/F	L/mT	1
17	L/T	L/T	L/T	L/T	L/T	L/T	L/m&laT	3
18	Neg	Neg	R/O	R/F	R/F	Neg	R/mT	1
19	L/F	Neg	Neg	L/P	L/P	L/F	L/mT	1
20	L/li	L/li	L/li	L/T	L/T	L/Fli	L/T &L/mT	1
21	L/F	L/F	R/T	R/O	R/O	L/F	R/TO	1
22	R/F	R/F	R/F	R/F	R/F	R/F	R/mT	1
23	Neg	R/F	R/F	L/T	L/T	Neg	L/mT	1
24	R/F	R/F	R/F	R/T	R/T	R/F	R/F	3
25	L/li	R/T	R/T	L/T	L/O	L/li	L/mT	3

*L, left; R, right; T, temporal; F, frontal; Neg, no finding; P, parietal; O, Occipital; L &R, left and right; l, lateral; m, medial; li, limbic; s-l, sub-lobar*.

**Figure 5:**
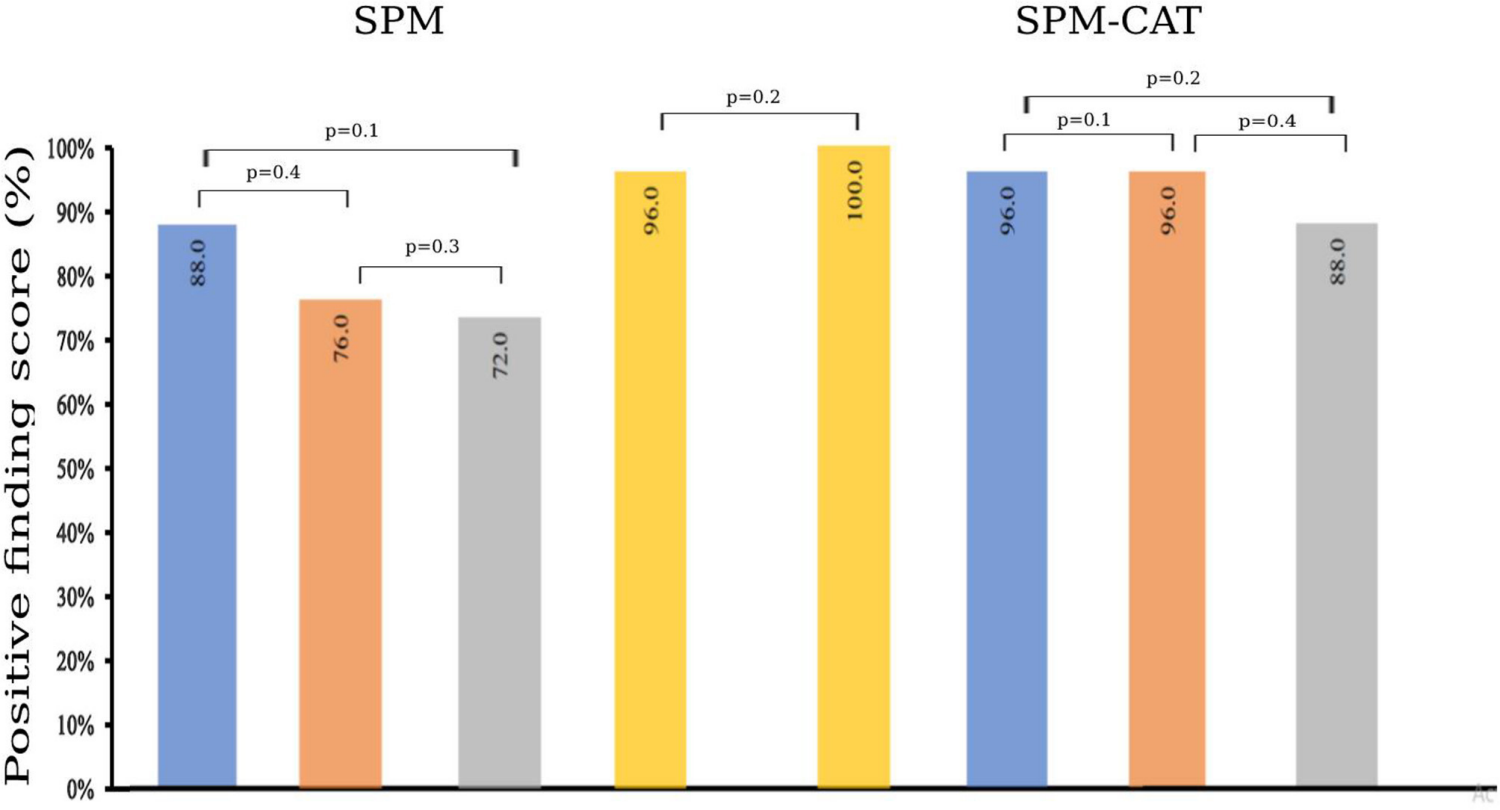
Correct localization percentage of SPM and SPM-CAT with different parameter settings (*p*-value is from McNemar's test and ^*^ means *p* < 0.05).

To compare the identified EZ by SPM and SPM-CAT with different settings, we obtain the corrected localization percentage, as given in Figure 5. For SPM, the correct localization percentage is 20.0% (5/25) for all the three settings (*p* < 0.0002, *k* > 25; *p* < 0.001, *k* > 100; *p* < 0.005, *k* > 200). The overall correct localization of SPM is 28.0% (7/25) and is significantly higher than that of the three settings (*p* < 0.05, McNemar's test).

The correct localization percentage of SPM-CAT with the three settings is 56.0% (14/25), 48.0% (12/25), and 20.0% (5/25), respectively. For each setting, SPM-CAT has a significantly higher correct localization percentage than SPM. SPM-CAT has obtained an overall correct localization percentage of 64.0% (16/25), which is significantly higher than that of SPM (*p* < 0.05, McNemar's test).

### Concordance Between the Identified EZ by SPM and SPM-CAT and the Confirmed EZ

Table 5 shows the agreement between the identified EZ with SPM and SPM-CAT with different parameter settings and the confirmed EZ according to the postsurgical follow-up. For SPM, *k* is 0.04 for the first setting (*p* < 0.0002, *k* > 25) and lower than that of the other two settings (0.1 and 0.5). In contrast, *k* is 0.5 for the first setting and higher than that of the other two settings (both 0.3) for SPM-CAT. The overall concordance of SPM-CAT is moderate (*k* = 0.5, CI 95% = 0.3, 0.7) while SPM is fair (*k* = 0.22, CI 95% = 0.06, 0.4).

**Table 5:** Concordance between the identified EZ by SPM and SPM-CAT and the postsurgical EZ.

	**Settings**	**Kappa index (*k*)**	**CI_**95%**_**
SPM	*p < 0.0002, k > 25*	0.04	0.2, 0.3
	*p < 0.001, k > 100*	0.1	0.1, 0.4
	*p < 0.005, k > 200*	0.5	0.3, 0.7
	*Overall*	0.22	0.06, 0.4
SPM-CAT	*p < 0.0002, k > 25*	0.5	0.3, 0.7
	*p < 0.001, k > 100*	0.3	0.1, 0.6
	*p < 0.005, k > 200*	0.3	0.07, 0.5
	*Overall*	0.5	0.3, 0.7

### The Number and Volume of Clusters Identified by SPM and SPM-CAT

**Figure 6:**
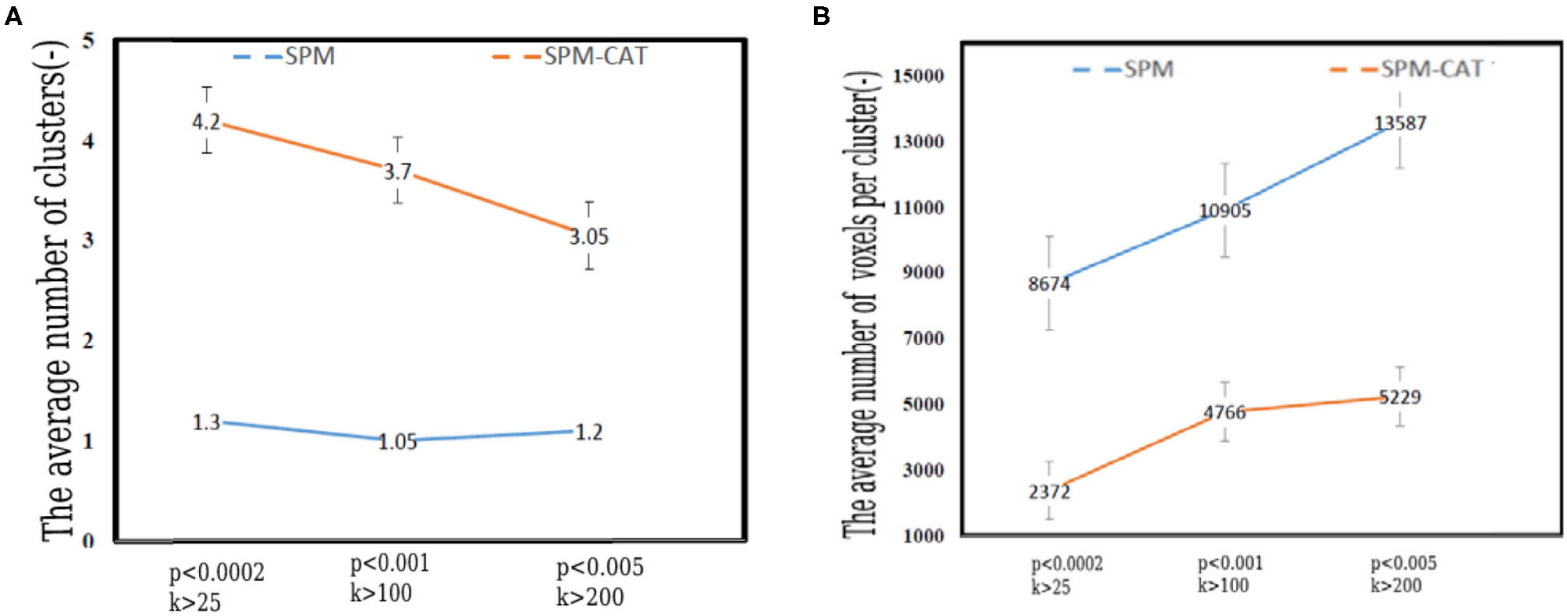
Number and volume of clusters identified by SPM and SPM-CAT with different parameter settings. **(A)** The average number of clusters. **(B)** The average volume of per cluster (number of voxels).

It is noted that one or more hypo-metabolic clusters can be found by SPM and SPM-CAT with different parameter settings. Therefore, the average number of hypo-metabolic clusters is presented in Figure 6A for each parameter setting (*p* < 0.0002, *k* > 25; *p* < 0.001, *k* > 100; *p* < 0.005, *k* > 200). The value for each setting identified for SPM-CAT (4.2, 3.7, and 3.05) is higher than that of SPM (1.3, 1.05, 1.2). The first setting (*p* < 0.0002, *k* > 25) identified more clusters than the other two settings. However, the average number of clusters is similar for three different settings in SPM.

Figure 6B shows the average volume (number of voxels) of the identified hypo-metabolic clusters of 25 epilepsy patients. For SPM, the average number of voxels per cluster is 8,674, 10,905, and 13,587 for the respective settings. This value is significantly higher than that of SPM-CAT: 2,372, 4,766, and 5,229. For both approaches, the highest value was obtained with the third setting (*p* < 0.005, *k* > 200).

## Discussion

### Utility of FDG-PET in the Identification of the Epileptogenic Zone

We assessed an FDG-PET data series from 25 subjects of a pre-surgical study lasting for more than a year that have been diagnosed with refractory epilepsy. Recent studies revealed that refractory epilepsy remains one of the most complicated cases of epilepsy in terms of its diagnosis (5). Several researchers have confirmed the utility of FDG-PET for the pre-surgical evaluation of refractory epilepsy patients, such as for CD. Salamon et al. conducted FDG-PET/MRI co-registration in their efforts to explore novel neuroimaging methods to detect cortical lesions (23). Their study's outcome has added value for the 33% of patients with no concordant EEG and neuroimaging findings. According to their study, the advantages of using the FDG-PET/MRI co-registration technique allowed for more precise surgical planning. The technique seemed to distinguish subtle lesions not appreciated by MRI or PET alone that turned out to be CD upon histopathological analysis.

As a functional neuroimaging method, PET can provide complementary information for patients who have normal MRI findings and require further intracranial investigation prior to surgery. Halac et al. performed a study to distinguish the compatibility of specific characteristics of FDG-PET analyses of FCD subgroups with MRI and clinical findings of the patients in these subgroups (7). Their study revealed that FDG-PET had demonstrated high sensitivity to hypo-metabolism in patients with refractory epilepsy and who had no findings in MRI results (MRI negative).

FDG-PET imaging plays an important role in the localization of epileptic foci. Tang et al. performed an investigation on kinetic parameters for epileptic foci identification (24). They assessed the correlation of parameters asymmetry indexes (ASYM) between dynamic and static FDG-PET to understand the hypo metabolism pathophysiology within intractable epilepsy. Dynamic FDG-PET provided an effective and complementary measure for epileptogenic zone detection in the small cohort for the authors and suggested that inter-ictal epilepsy was more impacted by glucose phosphorylation than by capillary influx.

FDG-PET functional imaging is likewise applied for localization of SOZ in epilepsy surgery. Elkins et al. present a gray-matter segmentation method for functional neuroimaging to localize SOZ in epilepsy surgery (25). They suggested that F-FDG-PET segmentation significantly increases the number of cases where an iEEG SOZ is correctly identified, often detecting an anatomically specific SOZ at the subgyral level.

### Voxel-Based Analysis

Accurately localizing epileptogenic zones remains challenging for medical scientists, and visual assessment is insufficient for most cases. Consistent and objective analysis methods must be employed to accurately localize the EZ.

This study proposes a VBM analysis to diagnose and localize the epileptogenic zone of 25 patients. Our assessment procedures followed a standard VBM pipeline as described in (13, 23). Our methodology consisted of comparing SPM to its toolbox CAT12 (SPM-CAT) associated with different parameter settings. The idea of using SPM and CAT12 toolbox to investigate refractory epilepsy is based on the need to improve visual analysis by accurately localizing the lesions zone and determining which VBM approach is best suited to make such analysis.

VBM demonstrated its effectiveness as a valuable method to investigate refractory epilepsy patients, as SPM and CAT12 have been widely used for this purpose. Mayoral et al. performed a study where the utility of SPM in PET-negative epilepsies was explicitly addressed (16). They demonstrated the usefulness of SPM with optimized thresholding in a series of 55 patients who underwent an FDG-PET study evaluated upon visual inspection, where 20 of 55 patients who had PET-negative studies had lesional MRI. The highest rate of positive and correctly localizing studies with SPM was obtained when the least restrictive threshold in *p*-value and the largest minimum cluster size were used. According to their study, SPM appeared to be offset by decreased specificity. Thus, they suggest that patients be accurately selected, and that PET must be requested when MRI alone is not sufficient to locate the SOZ with maximum certainty.

VBM can also be used to analyze brain activity, in particular brain changes associated with TLE. Chaudhary et al. also performed the evaluation of the semantic verbal memory outcomes in pre-and post-surgery TLE patients using functional MRI and voxel morphometric methods (26). VBM was applied using the statistical parametric imaging (SPM12) and CAT12 toolbox. Their study reveals a significant reduction in gray matter volume in the left temporal lobe, postoperatively compared to prenursery and healthy control groups. In the post-surgery TLE group, neuropsychological scores were reduced in specific PGI domains, such as visuospatial, working memory, and executive functioning.

### SPM or SPM-CAT for Positive Finding and Correctly Localizing EZ

The experiment in the present study provides novel insight into the relationship between SPM and SPM-CAT. Satisfactory results were obtained in terms of successful EZ localization. SPM and SPM-CAT achieved a positive localization percentage score of 96.0 and 100.0%, respectively. However, for individual parameter settings, a significant difference is observed. For both methods, the highest score was achieved with setting 1 (*p* < 0.0002, *k* > 25), while a lower score was achieved with (*p* < 0.005, *k* > 200). For a correct localization percentage, an overall score of 28.0 and 64.0% was achieved by SPM and SPM-CAT, respectively. For individual parameters settings, the highest score of 56.0% was achieved with setting 1 (*p* < 0.0002, *k* > 25) of SPM-CAT. However, this scenario was carried out slightly higher than the second scenario with a score of 48.0 and 20% achieved by other parameter settings (*p* < 0.001, *k* > 100, and *p* < 0.005, *k* > 200) for SPM-CAT. In contrast, SPM has achieved the same score of 20.0% for all three-parameter settings.

In our study, EZ is correctly localized by using SPM in only five out of 25 patients; for 10 out of the 25 patients, different positive regions are identified while changing the parameter settings of SPM. Such result highlights the motivation of our study, i.e., to explore an appropriate analysis method of EZ localization for better curative solution. Moreover, it has been well-known that only PET image analysis cannot accurately localize EZ for surgery, pre-operative SEEG and intraoperative ECoG must be used as the golden standard.

SPM-CAT shows higher sensitivity associated with the best performance and more correlation to the confirmed EZ according to the postsurgical follow-up. The main differences between these two VBM approaches might come from the pre-processing steps, where both approaches use different segmentations. SPM bases the image segmentation on tissue probability maps (TPM), which represents the prior probability of an image unit (voxel) being either gray or white matter or non-brain tissue (12). CAT12 uses TPM to normalize the image, perform an initial skull-stripping, and initialize the segmentation to update the estimation models for brain tissue classification and accounting for partial volume effects (27). Tavares et al. compared two segmentation pipelines (the SPM12 toolbox and an SPM12 add-on, the CAT12 toolbox) of structural brain MRI to investigate Alzheimer's disease (28). The authors suggested that SPM12 and CAT12 brain volume measure differences are tissue-dependent. The following steps are very relevant, in that (1) SPM12 volume estimates are strongly correlated with CAT12 volume estimates, while the absolute differences between pipelines are tissue specific; (2) pipeline choice modulates the effect of age on all volume measures and of diagnosis on hippocampi GM volumes computed from 3 T data; and (3) the pipeline has no effect on the accuracy of any brain volume measure detecting AD diagnosis.

CAT12 is a relatively novel tool that is computationally less expensive than SPM owing to its parallel processing algorithms. It enables more facilities in processing VBM and other processing methods. Farokhian et al. compared GM and WM abnormality results, obtained by VBM analysis using CAT12 via the current version of SPM12, with the results obtained by VBM analysis using the VBM8 toolbox implemented in the older software SPM8 (14). Their findings were consistent with the literature and pathology-based knowledge of VBM analysis using the TLE.

Comparing the performance of SPM-CAT to a previous study in (27), the PET-analysis obtained 66.7% (20/30) of correct localizations, which is comparable to our results (56.0%). Further, using CAT12, regional tissue volumes can be estimated in different regions based on the probabilistic atlases. However, further analysis must be conducted to confirm and improve this approach. The excellent performance achieved by the parameter setting 1 (*p* < 0.0002, *k* > 25) might come from the cluster's size, including the minimum size of the metabolic zone.

To measure the distance between the point of maximum VBM alteration and the center point of the surgically removed tissue can give more precise evaluation of the concordance between PET and surgery. However, the coordinates of center point of the surgically removed tissue are unknown for two reasons. First, this information is not recorded in the pre-operative plan and the surgically removed tissue might be changed according to measurement of the pre-operative SEEG and intraoperative ECoG. Second, it is difficult to localize the center point of the surgically removed tissue from the post-operative MRI images due to the potential deformation of brain. In the future, more advanced methods will be required and developed.

### Limitations and Future Works

The generalizability of the results has several limitations. Although the VBM automated approach has distinct advantages over conventional region-of-interest-based methods, it has certain limitations due to the source images' imperfect spatial normalization, segmentation, and smoothing. Volume differences in regions where none occur, such as gray matter changes in brain regions that should be white or gray matter, may result from systemic misclassification of structures (29). This limitation is common to SPM and SPM-CAT, while both techniques apply VBM. Further research is needed to assess this error; other methods such as surface-based morphometry (SBM) or tensor-based morphometry (TBM) can be explored to solve some of these issues. In the near future, multi-modality MRI including diffusion-weighted imaging and resting-state functional MRI (rs-fMRI) will be used to identify the potential regions and connections related to epileptogenic zone (30–32).

Another limitation of VBM analysis is about hypo-metabolic selection criteria. A common practice is to select the area with the largest volume identified by SPM and SPM-CAT as the EZ. One or more areas of reduced metabolism in PET could be caused by some reasons other than epilepsy, such as other neurological lesions, antiepileptic therapy, or functional alterations secondary to epilepsy (e.g., cognitive disorders). The identified clusters (or areas) caused by neurological lesions visible in MRI images can be excluded. However, all the patients in our study were MRI-negative. For hypo-metabolic clusters caused by other reasons, no good identification method is available. This limitation is mostly common to VBM applications; most previous studies have noticed this problem but they employed the same procedure as we did. Meanwhile, for the similar reason given above, as it is a very common procedure, the positive finding score defined in our study might be overestimated.

## Conclusion

SPM and SPM-CAT with different parameter settings can be employed to objectively detect the hypo-metabolic areas in FDG-FET images for refractory epilepsy patients. SPM and SPM-CAT have achieved the same overall positive finding score. However, according to different parameter settings, the positive finding score was different. SPM-CAT has achieved a higher positive finding score than that of SPM for each setting, which makes SPM-CAT more efficient than SPM in localizing EZ for refractory epilepsy by quantitative analysis of FDG-PET images. Moderate agreement is found between the confirmed EZ and the pre-surgical EZ identified by SPM-CAT. SPM-CAT with the setting of *p* < 0.0002 and *k* > 25 might perform as an objective complementary tool for the visual assessment of EZ localization.

## Data Availability Statement

The datasets presented in this article are not readily available because of the requirement of the Ethics Committee of Shengjing Hospital of China Medical University (Shenyang, China). Requests to access the datasets should be directed to Han Li, leoincmu@gmail.com.

## Ethics Statement

The studies involving human participants were reviewed and approved by Shengjing Hospital of China Medical University. Written informed consent to participate in this study was provided by the participants' legal guardian/next of kin.

## Author Contributions

EB performed experiments and analyzed the data along with SQ and CJ. SQ, DH, and HL conceived the study, presented the results, and wrote the manuscript along with EB. SH and LW collected and analyzed the data. SQ and LW supervised the algorithm development and analyzed the data. All authors read and approved the final manuscript.

## Funding

This work was partly supported by the National Natural Science Foundation of China (Grant Nos. 82072008, 81671773, and 61672146) and the Fundamental Research Funds for the Central Universities (Grant Nos. N2124006-3).

## Conflict of Interest

The authors declare that the research was conducted in the absence of any commercial or financial relationships that could be construed as a potential conflict of interest.

## Publisher's Note

All claims expressed in this article are solely those of the authors and do not necessarily represent those of their affiliated organizations, or those of the publisher, the editors and the reviewers. Any product that may be evaluated in this article, or claim that may be made by its manufacturer, is not guaranteed or endorsed by the publisher.

## Footnotes

^1^http://brainmap.org/training/BrettTransform.html.
